# Supplementary material for: The effect of malnutrition on adult Covid-19 patient’s ICU admission and mortality in Covid-19 isolation and treatment centers in Ethiopia: A prospective cohort study
Source: PLoS One. 2024 Mar 20;19(3):e0298215. doi: 10.1371/journal.pone.0298215 (PMC10954135; doi:10.1371/journal.pone.0298215)
Supplement: S1 Table — (DOCX) [file pone.0298215.s002.docx]

S1 Table: Socio-demographic characteristics of COVID-19 patients in SPHMMC, EKGH and MCCC, Addis Ababa, 2021

| Variables (N=581) | Frequency | Percentage |
| --- | --- | --- |
| Age Group |  | |
| <65 | 407 | 70.1% |
| >=65 | 174 | 29.9% |
| Sex |  |  |
| Male | 346 | 59.6% |
| Female | 235 | 40.4% |
| Residence |  |  |
| Urban | 527 | 90.7% |
| Rural | 54 | 9.3% |
| Marital status |  | |
| Single | 86 | 14.8% |
| married | 495 | 85.2% |
| living arrangement |  | |
| Alone | 97 | 16.7% |
| With a partner | 16 | 2.8% |
| With a parent/s | 468 | 80.6% |
| Income |  | |
| 0-600 | 100 | 17.2% |
| 601-1650 | 26 | 4.5% |
| 1651-3200 | 104 | 17.9% |
| 3201-5250 | 161 | 27.7% |
| 5251-7800 | 87 | 15.0% |
| 7801-10900 | 79 | 13.6% |
| >10,900 | 24 | 4.1% |
