# Supplementary material for: The effect of malnutrition on adult Covid-19 patient’s ICU admission and mortality in Covid-19 isolation and treatment centers in Ethiopia: A prospective cohort study
Source: PLoS One. 2024 Mar 20;19(3):e0298215. doi: 10.1371/journal.pone.0298215 (PMC10954135; doi:10.1371/journal.pone.0298215)
Supplement: Attachment — Submitted filename: A rebuttal letter Response to reviwers.docx [file pone.0298215.s003.docx]

A rebuttal letter

Dear PLOS ONE's Journal Editor

We would like to express our gratitude for giving us another opportunity to revise the manuscript as per reviewer comments. We affirm that the manuscript has improved a lot from your comments.

We hereby listed Point-by-point responses within the 'Response to Reviewer'.
Kind regards,

**'Response to Reviewers**

**Editor Comments:**

1. Please ensure that your manuscript meets PLOS ONE's style requirements, including those for file naming. The PLOS ONE style templates can be found at https://journals.plos.org/plosone/s/file?id=wjVg/PLOSOne_formatting_sample_main_body.pdf and https://journals.plos.org/plosone/s/file?id=ba62/PLOSOne_formatting_sample_title_authors_affiliations.pdf

***Address:*** *Dear reviewer, Thank you for the comment. We have corrected it as* *recommended. We have checked carefully to ensure the PLOS ONE’S requirements.*

2. Thank you for stating the following financial disclosure:

State what role the funders took in the study. If the funders had no role in your study, please state: “The funders had no role in study design, data collection and analysis, decision to publish, or preparation of the manuscript.”

**Address:** *Dear reviewer, Thank you for the comment. We have corrected it as recommended (line number 333)*

**Additional Editor Comments:**

**Reviewer comments**

Reviewer #1: The authors present relevant and unique data on associations between various forms of malnutrition and severe outcomes (ICU and/or death) among patients presenting to a single Ethiopia referral hospital with a Covid-19 diagnosis.

Several major concerns need to be addressed.

1. Malnutrition is a composite category, and the authors need to be clear and consistent. In this paper, both under nutrition and overweight were assessed, but not other kinds of malnutrition (eg micronutrient deficiencies). This is not a problem, but clear definitions need to be given by BMI and/or MAUC, and both types of malnutrition must not be lumped anywhere, when describing associations or assessing risks. These are different populations.

***Address:*** *Dear reviewer, Thank you for the comment. There was clear operational definition for malnutrition using BMI & MUACwith the help of ref [15].Undernourished: COVID-19 case with BMI <18.5 kg/m^2^and for those critically ill patients who are not capable of measuring BMI the MUAC measurement of<23.7 were considered as underweight (15). Overnourished:COVID-19 case with BMI ≥25 kg/m^2^and for those critically ill patients who are not capable of measuring BMI the MUAC measurement of* $\geq$*28.1were considered as overweight (15).*

2. BMI>25 and in particular BMI>30 are already well-established risk factors for adverse outcomes following Covid-19 infection. Thus, the added value of this paper is more in showing, or contrasting the risks associated with undernutrition. This would give a more clear perspective to introduction and discussion, and the paper overall.

***Address:*** *Dear reviewer, Thank you for the comment. The add value of this research paper is more on showing the adverse effect of undernourished on COVID-19 patients. As you suggested it is elaborated in detail in discussion section of the paper.*

*3.* The authors need to present a more thoughtful discussion on implications of their findings, beyond providing unclear statements on 'suitable interventions to be provided'. It is not clear what interventions they want to see implemented, when, why, nor to what extent there are evidence-based interventions, in particular upon hospitalization, which improve outcomes assessed. Are they suggestion to put obese patients on a strict diet&exercise plan? Force-feeding undernourished patients?

***Address:*** *Dear reviewer, Thank you so much for your valuable comments.The study emphasizes the importance of early nutritional screening and to promote sufficient attention on nutritional intervention through developing guidance for covid-19 nutritional management.*

4. Also, it is remarkable that some risk factors observed in other settings (co-morbidities, age) were not significantly associated with adverse outcomes here. This can cast doubt on the validity of the data collected and conclusions reached, and needs at least some thoughtful reflection.

***Address:*** *Dear reviewer, Thank you for your insightful comment, our research haven’t revealed any association between co-morbidity and adverse COVID-19 this might be due to the sample size was calculated based on the proportion of adverse effect of COVID-19 patient among the malnourished and well-nourished patients.*

5. In contrast to many other underlying conditions, manifest undernutrition and overnutrition are not very difficult to assess visually by trained staff, thus the statement that these condition are often unrecognised is unclear. Perhaps the authors meant that the associations with of increased risk for adverse outcomes are not always appreciated, but either way it would be good to have references to back up such unexpected claims.

***Address:*** *Thank you again for your valuable comments.* *Saying left unrecognized is an ambiguous phrase so we have to change into “doesn’t gate enough attention”this can explain it well. Besides there is a literature which suggests Malnutrition in developing countries including Ethiopia tends to refer to children and awareness about adult disease-related malnutrition is lack, so it stills a major problem in a clinical setting. Because of lack of knowledge, routine nutrition screening, assessment and intervention practices are not uniformly done as a part of medical care (21)*

6. More information needs to be given on the Covid-19 policies in the setting. Were these all patients referred, or were they a mix of direct presentations and referrals? Were all patients included because of their Covid-19 related clinical state, or were there also patients included who presented with an unrelated problem, but were routinely tested and found infected?

***Address:*** *Dear reviewer, Thank you so much for your valuable comments. The centers selected for the study was designated for covid-19 patients treatment only. So the other health institution in the city can referred covid-19 cases to these centers for isolation, well-equipped ICU setup and for their larger admission capacity.But some referred caseswere found while routine covid-19 tests.*

7. The presentation of data needs to be much improved. Baseline data on BMI and MUAC need to be presented by outcome category. Clarification needs to be given to what extent deaths occurred at ICU or elsewhere, and how data on patients who were first admitted to ICU, and later died (either at ICU or in the ward were analyzed. Were there cases where patients opted to go home to die? While the KM curves provide some key information on time to events, it does not clarify patients with both events, and these findings are not properly mentioned in the text. Are there background data on the prevalence of obesity and undernutrition in the catchment populations?

***Address:*** *Dear reviewer, Thank you so much for your valuable comments.There is no case consideration of ICU admission after death outcome .For further clarification those patients admitted to ICU, and later died while follow up were reported as death outcome.ICU admission and death was censored at 2 months, hence COVID-19 patients who were discharged earlier than follow up time without adverse outcome or stayed in the hospital for more than 2 months were treated as censored observations. There was no any background data on the prevalence of obesity and undernutrition in the catchment population.*

8. The authors also need to reflect on potential non-causal explanations for the association between undernutrition and adverse outcomes. Eg, is it possible that undernourished people have less easy access to health services (eg due to poverty, rural residence, civil unrest, with more time to reach/less available health services, etc), and might they therefore be presenting at a more severe or later stadium, resulting in worse outcomes? Was there information available on distance of the residence to the hospital, and on duration of complaints prior to arrival? How many critically ill (defined as? Assessed by someone indepent from the study team?) Were excluded?

***Address:*** *Dear reviewer, Thank you so much for your valuable comments. It is bit difficult to reflect some of the factor which may have effect on the association of under nutrition and adverse outcome because of during base line data collection the mentioned factors weren’t included in the study. We didn’t include the onset of the disease because the patients were enrolled based the severity of the disease at admission.Individual who full fill the exclusion criteria they haven’t been registered by the data collectors. So there is no compiled number which shows the number of excluded patients who are critically ill at admission.*

9. Please clarify why MUAC was used in addition/as alternative to BMI, as inclusion was restricted to adults, and critically ill patients for whom nutritional status could not be assessed at admission were excluded already?

***Address:*** *Dear reviewer, Thank you so much for the comment. Wrongly wrote the sentence in line 88 as we all know MUAC measurement was need to measure critical ill patients who are not capable of measuring their BMI. So we put the correct sentence 'critically ill patients with both upper extremities amputation were excluded’.MUAC is also recommended for patients with ascites or edema in legs or trunk to gauge dry weight and BMI(16).Other studies confirm that MUAC correlated well with BMI and could be used to identify patients as underweight and overweight(17-20).*

10. A major overhaul is needed to reduce/eliminate the frequent repetitions and superfluous sentences where no relevant information is presented; also in part of the methods there is an overload of details relating to standard procedures, which is unnecessary; and in parts of the results there is quite a bit of repetition of non-essential data already presented in the tables/figures, rather than highlighting the main findings only in the text.

*Address: Dear reviewer, Thank you for the comments. We agree with you sir. So we have addressed those comments.*

Some minor comments:

1. The Abstract mentions in the results that the mean age was 55 years, (16,45) years. Please clarify the 16,45 (also in the Results section).

***Address:*** *Dear reviewer, Thank you for the comments. The sentence in line 166 show that the mean age of study sample was 55 years and the standard deviation was 16.45 years.*

2. Methods: it seems a bit odd to justify the measurements referring to a (non-peer reviewed) publication related from the Australian sports federation (ref 14). Is that a relevant context to provide validity to an Ethiopian hospital setting.

***Address:*** *Dear reviewer, There is no study which shows acceptable TEM level in Ethiopian hospital setting.*

3. Methods: with a mean age of 55, it is surprising that 81% were were categorised as living with parents, and less than 3% with a partner. Presumably, elderly parents might be living with their grown up children, with or without partner? However, in general it is not clear what the added value of this category is, suggest to drop.

***Address:*** *Dear reviewer, Thank you for the comments. Yes it was confusing, so we have to omit the sentence in line 173-175 general.*

4. Results: unclear why ages were split in only 2 categories, please do at least a sensitivity analysis with eg 4 categories. On the other hand, 7 categories for income do not seem useful, suggest to reduce to eg 4.

***Address:*** *Dear reviewer, Thank you for the comments. From earlier research adults over 65 years of age represent 80% of hospitalizations and have a 23-fold greater risk of death than those under 65.this is the main reason for age categorization.*

5. For smoking and alcohol: clarify if this refers to current situation or ever. The high number of people taking over 5 medications (60%) appears at odds with 48% reporting no co-morbidity, can you explain? More information on what co-morbidities might be helpful.

***Address:*** *Dear reviewer, Thank you for the comments. The substance status refers to ever smoking and alcohol drinking status. Even if larger number of covid-19 patients doesn’t have co-morbidity covid-19 disease by itself need supportive medications/supplements like vitamin A, C, zinc and iron as a result they take over 5 medications.*
