# Supplementary material for: The effect of malnutrition on adult Covid-19 patient’s ICU admission and mortality in Covid-19 isolation and treatment centers in Ethiopia: A prospective cohort study
Source: PLoS One. 2024 Mar 20;19(3):e0298215. doi: 10.1371/journal.pone.0298215 (PMC10954135; doi:10.1371/journal.pone.0298215)
Supplement: Attachment — Submitted filename: Response to Reviewers.docx [file pone.0298215.s004.docx]

A rebuttal letter

Dear PLOS ONE's Journal Editor

We would like to express our gratitude for giving us another opportunity to revise the manuscript as per reviewer comments. We affirm that the manuscript has improved a lot from your comments.

We hereby listed Point-by-point responses within the 'Response to Reviewer'.
Kind regards,

*N.B, the line number we mentioned here is based on the revised track change manuscript file

**'Response to Reviewers**

**Reviewer comments**

Reviewer #3:

1. Line 54: Correct "SARS-Cov2 infection (COVID-19) and related pneumonia" to "SARS-CoV-2 infection and related pneumonia (COVID-19)"

**Address:** *Dear reviewer, Thank you for your valuable suggestion. We have duly revised the statement in accordance with your guidance.* *(Page 3, line number 54).*

2. Line 85: "All COVID-19 cases with confirmed positive COVID-19 test result were included..." What type of SARS-CoV-2 tests were used, eg. RT-PCR or antigen? The line should read SARS-CoV-2 test rather than COVID-19 test. How were patients with SARS-CoV-2 infection differentiated from those who specifically had COVID-19 pneumonia?

**Address:** *Thank you for bringing this matter to our attention in the main research paper we have addressed the issue by explicitly stating that we utilized the rRT-PCR test for diagnosing the SARS-CoV-2 virus. This clarification has also been incorporated into the track change document for your reference. (Page 5, line number 89-91).*

3. Line 86: "Patients were excluded from the study if they are critically with both upper extremities amputation ill..." Correct this sentence for meaning.

**Address:** *Dear reviewer, Thank you for your valuable suggestion. We have revised the sentences to enhance clarity and meaning, aligning with your feedback (page 5, line number 92-94)*

4. Line 99: "Based on this assumption, a total of 581 COVID-19 patients were included in the study based on the highest sample size obtained from ICU admission prevalence." As a prospective study, it would be better to state that 581 COVID-19 patients were required rather than included.

**Address:** *Thank you for your valuable suggestion. In response, we have refined the statement to convey a more accurate representation. Instead of stating that 581 COVID-19 patients were included, we now specify that this number was required for the study based on the highest sample size obtained from ICU admission prevalence (page 5, line number 105)*

.

5. Line 128: "MUAC measurement of<23.7 were considered" Provide unit of measurement for MUAC each time it is used.

**Address:** *Thank you for bringing this matter to our attention. We have addressed the issue, and the corrected measurement is now recorded as 23.7 cm. (page 6, line number 134)*

6. Line 177: "There were statistically significant association seen between age, sex, income, co morbidity, medication and nutritional status." This sentence could be more specific, e.g. "There were statistically significant association seen between nutritional status and younger age, female sex, income, co morbidity, and higher number of medications."

**Address:** *Dear reviewer, Thank you for your suggestion. We have refined the sentence to specify a statistically significant association between nutritional status and younger age, female sex, income, co-morbidity, and a higher number of medications (page 9, line number 191-192).*

7. Line 178: "More percentage of patients < 65 years age group was seen in both malnourished (77.9%) and well-nourished 182 (62.3%) nutritional status." This statement is non-contributory as it only means more patients aged < 65 we recruited compared to > 65. It would be more helpful to give the percentage of patients under and over 65 who were well nourished and malnourished (i.e. use the number of patients in each age group as the denominator) to enable comparisons.

**Address:** *Thank you for your constructive comment. In response, we have made the suggested revision and incorporated it with track changes for your review. (Page 9, line number 192-195).*

8. Line 179: "Malnutrition of male patients was higher than female in the cohort (50.9% vs 49.1%, p<0.001)." It is true that the absolute number of male patients who were malnourished is higher, but the proportion of female patients who are malnourished is actually higher (42% vs 60%). The authors have again used the incorrect denominator. The same is true for the other comparisons made in this paragraph.

**Address:** *Thank you for highlighting this issue. We have addressed it by making the recommended revision, and you can find the changes incorporated in the track changes for your review (Page 9, line number 196-197).*

9. Line 184: "From preexisting medical conditions diabetic mellitus (31.32%) is the most common cause of co-morbidity then hypertension (25.6%), other diseases (17%), lung diseases (9.8%)..." Consider including specific comorbidities in table 1, divided by nutritional status. Several of these comorbidities are known to be risk factors for poor outcomes in COVID-19 and their effects on the findings of this study need to be considered.

**Address:** *Dear reviewer, Thank you for your comment. We have addressed the feedback by incorporating the listed co-morbidities into Table 1 (Page 11-12).*

10. Line 195: "Nutritional status and clinical outcome among COVID-19 Patients". It is difficult to assess the accuracy of the statistics provided here as no absolute numbers are provided. It is also not stated how many patients were overweight. Overweight patients seem to be separated in some places, and grouped together with well-nourished patients in others, such as the preceding paragraphs and table 1. The authors need to be more consistent with how patients are grouped.

**Address:** *Thank you for your comment. The absolute numbers of both malnourished and well-nourished cases have been specified as recommended (Page 9, line number 189-190).*

11. Line 263: "Of these, 211(40.3%) were admitted to the ICU as a result of lower BMI (23)." More accurate to state that they had lower BMI, not that the admission was as a result of the lower BMI.

**Address:** *Dear reviewer, Thank you for your comment. We have rephrased the statement according your suggestion.* *(Page 18, line number 291-292).*

12. Line 264: "Every study conducted in different". Numerous studies, but not every study. Many studies did not report underweight patients.

**Address:** *Dear reviewer, your comment is absolutely right and we have corrected it as recommended (Page 18, line number 293-294).*

13. Figure 1: The percentages only add up to 66.68%. What happened to the remainder?

**Address:** *Dear reviewer, figure 1 only shows the percentage of ICU admission and death among malnourished and well-nourished patients, out of the total COVID-19 patients enrolled. The remaining were neither admitted to the ICU nor died. (The figure only shows patients with an outcome)*

**Reviewer #4**

1. The introduction needs some minor language editing.

**Address:** *Dear reviewer, we have revised the whole manuscript for spelling and grammar*.

2. The authors dictated they have conducted a prospective cohort despite this unusually they have 0% drop out. Furthermore, the authors follow the patients until 2 months since the follow-up seems long there was no withdrawal which is impractical.

**Address:** *Dear reviewer,* *your comment is absolutely right and we understand your concern. During the initial days of the COVID-19 pandemic in Ethiopia, a limited number of isolation and treatment centers were available, wherein patients underwent isolation for several weeks. The isolation duration extended until two consecutive rRT-PCR tests returned negative results, at which point patients were considered cleared. This regulatory requirement compelled patients to remain in isolation until the manifestation of the desired outcome. Consequently, there was no challenge in maintaining follow-up within our cohort.*

3. Since the study was prospective cohort all 581 patents were followed for 2 months but was there a patient died being admitted to ICU? The authors should perform a sub-group analysis for malnutrition and death based on ICU admission status.

**Address:** *Thank you for your insightful comment, we did not include cases where ICU admission occurred after the patient's demise in our analysis of death outcomes. For additional clarification, instances involving patients admitted to the ICU who subsequently passed away during the follow-up period were classified and reported as death outcomes.*

4. How many patients were admitted to ICU and died during the follow-up? How the authors treat those patients defer admission to ICU but decided to be admitted by the attending physician? Why patients were followed for 2 months? Was there any justification?

**Address:** *Dear reviewer, Thank you for identifying this flow. In our efforts to rectify the issue, we have revised the statement to provide a clearer depiction of the overall mortality and subcategories based on nutritional status. The total number of deaths recorded during the study period was 16, comprising 9 individuals classified as overweight, 6 identified as undernourished, and 1 classified as well-nourished. Notably, all patients who succumbed had been admitted to the Intensive Care Unit (ICU) prior to their demise.*

*This period allows for monitoring the protracted recovery period and identifying persistent symptoms like fatigue and neurological issues. Additionally, it enables the assessment of severe outcomes such death and ICU admission. On top of this period allows us to identify long-term complications, including those emerging weeks after recovery.*

5. The authors should cite paper for the assumption used for double population formula.

**Address:** *Dear reviewer, the assumptions regarding sample size were initially included in the main document, but for manuscript purposes, they were omitted. However, in the current version, these assumptions have been reintroduced and appropriately cited. The basis for determining the prevalence of ICU admission and death among COVID-19 patients was derived from articles 31 and 32 (Page 24, line number 454-458).*

6. Statistical analysis: A survival analysis model was used to determine whether there was association between malnutrition and adverse Covid-19 outcome. However proper statistical procedures were not employed. The dependent variable should be time to event (event= admission to ICU or death). I strongly Did the authors check the proportional hazard assumption (using Schonefeld residual or log-log plot) before fitting the regression model? If so, it should have to be reported.

**Address:** *Dear reviewer, Thank you for your insightful comment. In our analysis conducted using SPSS software, both Schoenfeld residuals and log-log plots were employed to assess the proportional hazard assumption, specifically examining the parallelism between survival curves for the variables of interest. This comprehensive evaluation aimed to ensure the constancy of hazard ratios over time. The results of these diagnostic checks are duly reported in our study as recommended (Page 8, line number 159-161).*

7. The results were a little confusing the authors should present 1. The baseline characteristics 2. The exposure and outcome tabulation 3. The regression model results. The figures showing the Kaplan-Meier survival curve are not visible.

**Address:** *Thank you for your valuable feedback. While the baseline characteristics and regression details were initially lacking elaboration in the manuscript, I want to highlight that the exposure and outcome tabulations, which were previously omitted, have now been incorporated in the latest version. This addition enhances the comprehensiveness of the manuscript and addresses the noted omission.*

8. The result of Cox-regression model ton assesses the effect of malnutrition on Covid-19 mortality shows there was no difference between the crude and adjusted hazard ratios this indicate the variable (Malnourished/ Well-nourished) was not confounded by other variables this indicated the baseline characteristics were similar which is unlikely and results to be very questionable.

**Address:** *Dear reviewer, Thank you for your valuable comment. The observed similarity in the crude and adjusted hazard ratios concerning death reports among well-nourished and malnourished patients can be attributed to the limited number of recorded deaths during the study period. Specifically, the occurrence of only one death within the well-nourished cohort may have led to statistical challenges and influenced the stability of hazard ratio estimates.*

9. This may be clearer to the reader if it adhered more closely to a standard four paragraph structure: Para 1: summaries main findings, Para 2: compare and contrast with previous work, Para 3: strengths and limitations, Para 4: conclusions and future work. The authors cover most of this already but I think the limitations could be more detailed.

**Address:** *Dear reviewer, Thank you for your valuable input. In response to your guidance, we have made efforts to concisely address the limitations within the discussion section, adhering to the goal of brevity as suggested.*
